# Effect of glycemic control on soluble RAGE and oxidative stress in type 2 diabetic patients

**DOI:** 10.1186/1472-6823-13-32

**Published:** 2013-08-21

**Authors:** Tarek MK Motawi, Mohamed A Abou-Seif, Ahmed MA Bader, Mohamed O Mahmoud

**Affiliations:** 1 Department of Biochemistry, Faculty of Pharmacy, Cairo University, Cairo, Egypt; 2 Department of Internal Medicine, Faculty of Medicine, Beni-Suef University, Beni-Suef, Egypt; 3 Department of Biochemistry, Faculty of Pharmacy, Beni-Suef University, Beni-Suef, Egypt

**Keywords:** sRAGE, Oxidative stress, HbA_1c_, Type 2 DM

## Abstract

**Background:**

The interaction of advanced glycation end products (AGEs) and its receptor (RAGE) has played an important role in the pathogenesis of diabetes and its complications. A soluble form of RAGE (sRAGE) has been reported as a decoy receptor for AGEs. Oxidative stress is demonstrated in pathological condition such as atherosclerosis and diabetes mellitus. It has been suggested to be involved in the pathogenesis of both macro- and microvascular complications. This study was designed to evaluate the effect of glycemic control on sRAGE and oxidative stress markers in type 2 diabetic patients.

**Methods:**

Seventy patients with type 2 diabetes and 20 healthy subjects were recruited into the study. Blood glutathione (GSH) and plasma total nitric oxide (NO_x_) levels were measured using commercially available colorimetric kits, blood superoxide dismutase (SOD) activity was measured by the method of Marklund and Marklund, and plasma C-peptide, oxidized LDL (ox-LDL), sRAGE, and VCAM-1 levels were measured using competitive ELISA kits.

**Results:**

Plasma sRAGE levels were significantly lower (*p* < 0.05) while VCAM-1 levels were significantly higher (*p* < 0.05) in poorly controlled diabetic patients compared with healthy control. Blood GSH levels were significantly lower in diabetic patients compared with healthy control (*p* < 0.05). Plasma C-peptide, NO_x_, ox-LDL levels, and SOD activity were not significantly different in diabetic patients compared with healthy control. Plasma levels of sRAGE were negatively associated with circulating VCAM-1 levels in diabetic patients.

**Conclusion:**

Poor glycemic control decreases plasma sRAGE and increases VCAM-1 levels while good glycemic control improves these abnormalities which provides benefit to diabetic patients.

## Background

Diabetes mellitus (DM) is a complex disease with many metabolic disorders characterized by hyperglycemia and defects in insulin secretion or insulin action [1]. Type 2 diabetes mellitus (T2DM) is a global epidemic that increases in a very rapid way to reach over 300 million by the year 2025 [2].

The formation of advanced glycation end products (AGEs), by the so called Maillard reaction, is a complex process of condensations, rearrangements, fragmentations, and oxidative modifications which leads to a complicated network of heterogeneous products [3]. Interaction of AGEs with their cellular receptors (RAGE) has an import-ant role in the pathogenesis of diabetic complications *via* enhanced expression of adhesion molecules, including vascular cell adhesion molecule-1 (VCAM-1) and intercellular adhesion molecule-1 (ICAM-1), as well as increased vascular permeability and generation of procoagulant tissue factor [4]. The AGEs action can be neutralized by usage of soluble RAGE (sRAGE), the extracellular ligand-binding domain of RAGE, which competes with RAGE for binding AGEs. It was reported that administration of sRAGE in diabetic animals following induction of diabetes suppressed the vascular changes of accelerated diabetic atherosclerosis in these animals [5]. However, the AGE-RAGE interaction is still not clear. There may be more important ligand for RAGE than AGE-modified proteins physiologically. S100/calgranulin proteins, high mobility group-1 protein (HMGB1) and other proteins are candidate agonists *in vivo*[6].

Many studies reported increased levels of VCAM-1, one of the markers of vascular injury, in diabetes mellitus and might play a role in the changes observed in the vascular structure of diabetic patients [7,8]. The effect of high glucose levels on increasing adhesion molecules (ICAM-1 and VCAM-1) have been proved in many studies [9,10].

Oxidative stress is suggested as a mechanism underlying diabetes and diabetic complications. Enhanced oxidative stress and changes in antioxidant capacity, found in both clinical and experimental diabetes, are thought to be the main cause of chronic diabetic complications [11]. Excessively high levels of free radicals cause damage to vital cellular components such as proteins, membrane lipids, and nucleic acids, and finally lead to cell death [12].

Based on the above considerations, we hypothesized that tight glycemic control may restore plasma levels of sRAGE, VCAM-1 and oxidative stress parameters near the normal level in type 2 diabetic patients. The aims of the present study were (i) evaluate the effect of glycemic control on plasma levels of sRAGE, VCAM-1 and some oxidative stress markers (NO_x_, ox-LDL, SOD, GSH) in type 2 diabetic patients, (ii) determine the association between plasma sRAGE, parameters of oxidative stress (NO_x_, ox-LDL, SOD, GSH) and various relevant plasma factors (VCAM-1, C-peptide) as a function of glycemia in these patients.

## Methods

### Subjects

This study was conducted in the Out-patient Clinic of Beni-Suef University Hospital and comprised 90 subjects; 20 healthy control volunteers and 70 patients with type 2 diabetes. All patients submitted to the study underwent a full medical history, including age, duration of DM and body mass index (BMI). In addition, blood pressure measurement and a full medical investigation to screen for diabetic complications, the presence of cardiovascular diseases and arteriosclerosis were performed to all patients enrolled in the study. Electrocardiography and echocardiography were performed to all patients and to the control group that took part in this study. This study was approved by the local ethical committee of Beni-Suef University. Full informed consent was obtained from all participants in the study.

All patients enrolled in the study fulfilled the following inclusion criteria: age between 40–70 years; receiving stable antidiabetic therapy (sulfonylureas, metformin, and/or insulin) for at least 6–8 months and no history of ketoacidosis.

Exclusion criteria included the following: clinically significant neurological, hepatic, endocrinologic, or other major systemic diseases, such as malignancy, elevated plasma transaminases activity over twice the upper limit of normal, elevated plasma creatinine concentration (>1.7 mg/dl) [13], acute major cardiovascular events in the previous 6 months, presence of acute or chronic inflammatory diseases, and hormone replacement therapy for women subjected to the study. Exclusion criteria also included treatment with antineoplastic agents, psychoactive agents, glucocorticoids, statins, or vitamin supplements.

The patients enrolled in the study were classified into the following groups according to glycemic control; where the patients were subdivided into good glycemic control (HbA_1c_ ≤ 7.0%) and poor glycemic control (HbA_1c_ > 7.0%) [14] along with normal subjects: *Group 1* (control group) (C): included 20 age- and BMI-matched healthy individuals. *Group 2:* included 28 good controlled diabetics (GCD). *Group 3:* included 42 poorly controlled diabetics (PCD).

### Sample collection

Venous blood samples were withdrawn after overnight fast from each subject enrolled in the study. Each blood sample was collected into EDTA-treated tubes and divided into 2 aliquots. The first aliquot was used for estimation of blood GSH, glycated hemoglobin (HbA_1c_), and SOD activity. The second aliquot was centrifuged at 2000 × g for 10 minutes to obtain plasma which intern divided into 2 parts; the first part used for estimation of fasting plasma glucose (FPG) and creatinine levels, and AST and ALT activities while the second part was stored at −20°C for subsequent estimation of plasma C-peptide, sRAGE, VCAM-1, ox-LDL, and NO_x_ levels.

### Methods

Determinations of all the parameters were done using commercially available kits: plasma glucose was measured by glucose oxidase method (Spinreact, Santa Coloma, Spain). HbA_1c_ level was measured by cation exchange resin (BioSystems, Barcelona, Spain). AST and ALT activities were measured by kinetic methods (Spinreact, Santa Coloma, Spain). Creatinine was measured by Jaffé method (Diamond Diagnostics, Egypt).

GSH was measured by 5,5’-dithiobis(2-nitrobenzoic acid) (DTNB) method (Biodiagnostic, Giza, Egypt). Determination of blood SOD activity was based on the method of Marklund and Marklund [15]. Plasma NO_x_ was measured by Parameter™ total nitric oxide and nitrate/nitrite colorimetric assay kit (R&D Systems, Inc., Minneapolis, USA), plasma C-peptide was determined using ELISA kit (DRG®, Mountainside, N.J., USA), plasma VCAM-1 and sRAGE were determined using Quantikine® ELISA kits (R&D Systems, Inc., Minneapolis, USA), and plasma ox-LDL was measured by competitive ELISA kit (Immundiagnostik AG, Bensheim, Germany).

Spectrophotometric measurements were performed using Shimadzu Double Beam UV-spectrophotometer (Shimadzu, Japan); while ELISA readings were measured using Tecan Sunrise microplate reader (Austria).

### Statistical analysis

Data are presented as means ± SEM values. The results were analyzed statistically by one-way ANOVA with subsequent multiple comparisons using Tukey multiple comparisons test. Significance level was set at *p* < 0.05. Correlations between variables were assessed by Pearson’s correlation test. All calculations were made using the computer program SPSS 16.0 (SPSS, Chicago, Ill, USA). Power calculations between the 3 main groups were done using PS Power and Sample Size Calculations Software, version 3.0.43 for MS Windows (William D. Dupont and Walton D., Vanderbilt, USA).

## Results

Clinical characteristics and biochemical changes among the three studied groups are listed in Table 1. FPG and HbA_1c_ levels were significantly increased (*p* < 0.05) in GCD and PCD compared with C group. Moreover, HbA_1c_ levels were significantly increased in PCD compared with GCD.

**Table 1:** Clinical characteristics and biochemical changes among the three studied groups

**Parameter**	**Control healthy subjects (C) (20)**	**Good controlled diabetic patients (GCD) (28)**	**Poorly controlled diabetic patients (PCD) (42)**
Sex (M/F)	8/12	13/15	10/32
Age (years)	51.25 ± 1.51	56.39 ± 1.43	54.00 ± 1.17
BMI (kg/m^2^)	30.00 ± 0.82	31.72 ± 0.77	32.02 ± 0.52
Duration of diabetes (years)	—	7.82 ± 0.76	10.48 ± 1.01
SBP (mmHg)	120 ± 2	129 ± 2^a^	130 ± 2^a^
DBP (mmHg)	78 ± 1	82 ± 1^a^	81 ± 1^a^
FPG (mg/dl)	79.50 ± 3.07	155.11 ± 13.01^a^	191.02 ± 13.04^a^
HbA_1c_ (%)	5.26 ± 0.08	6.15 ± 0.10^a^	8.71 ± 0.57^a,b^
C-peptide (ng/ml)	4.09 ± 0.30	4.46 ± 0.25	4.13 ± 0.24
sRAGE (pg/ml)	804.92 ± 58.14	630.47 ± 48.14	600.06 ± 37.75^a^
VCAM-1 (ng/ml)	636.81 ± 30.33	739.69 ± 32.26	808.84 ± 36.90^a^
SOD (U/ml)	5.28 ± 0.40	7.44 ± 0.67	6.77 ± 0.61
NO_x_ (μmol/l)	177.08 ± 3.81	178.65 ± 4.04	187.15 ± 2.84
Ox-LDL (ng/ml)	94.67 ± 4.28	96.78 ± 6.59	114.31 ± 8.05
GSH (mg/dl)	45.81 ± 1.01	41.10 ± 1.34^a^	39.57 ± 0.98^a^
Antihypertensive therapy, *n* (%)	—	12(43)	22(52)

Values are means ± SEM, with the number of patients (*n*) given in parentheses.

^a^*p* < 0.05 *versus* control healthy group.

^b^*p* < 0.05 *versus* good controlled diabetic patients.

Using one-way ANOVA followed by Tukey post-hoc test.

sRAGE levels were significantly decreased (*p* = 0.01) while sVCAM-1 levels were significantly increased (*p* = 0.006) in PCD compared with C group. On the other hand, no significant change was observed in these parameters in GCD when comparing with C group.

No change was found in SOD activity, NO_x_, ox-LDL, and C-peptide levels in both diabetic groups when compared with each other or with C group. GSH levels were significantly decreased (*p* < 0.05) in both diabetic groups compared with healthy control.

**Figure 1:**
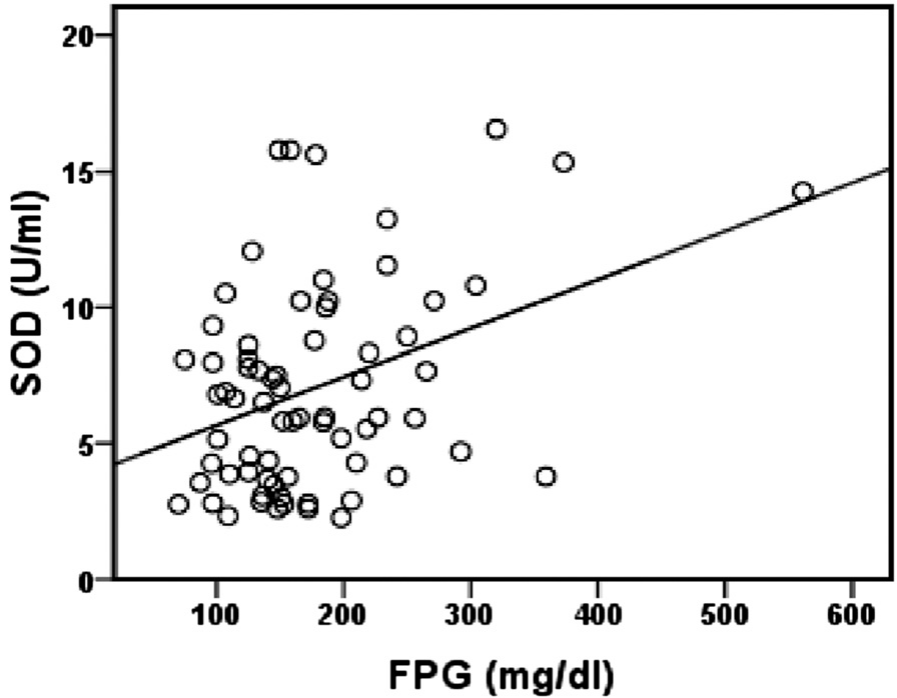
**Correlation between FPG level and SOD activity in T2DM patients (*r*** **= 0.377, *p*** **= 0.001, *n *=70).**

**Figure 2:**
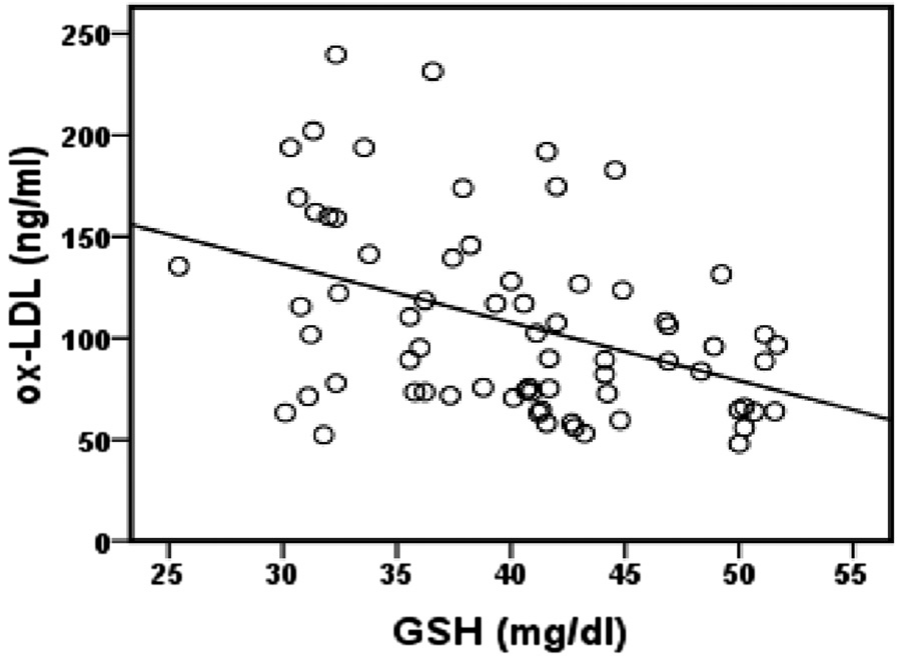
**Correlation between GSH and ox-LDL levels in T2DM patients (*r*** **= − 0.412, *p*** **= 0.00039, *n *=70).**

**Figure 3:**
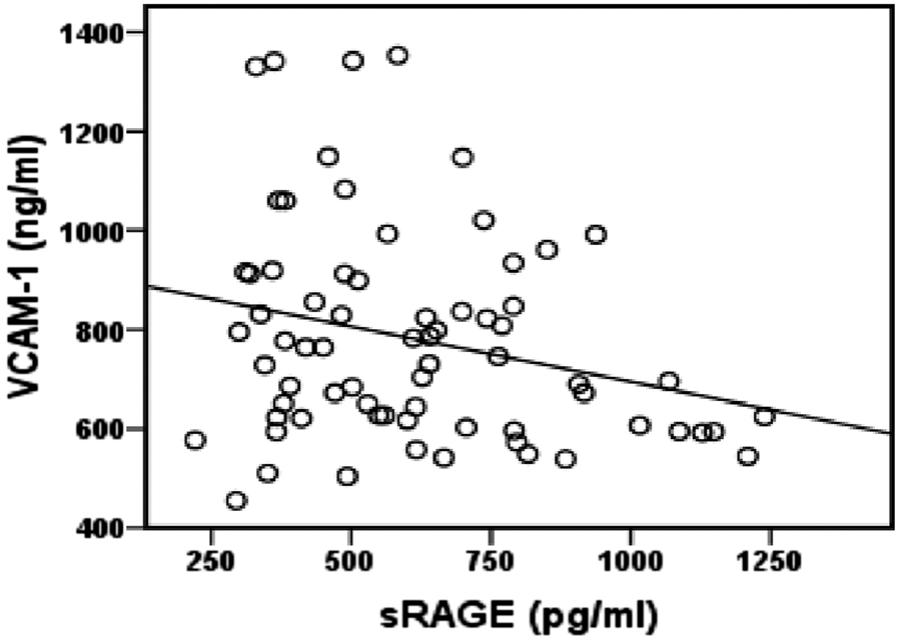
**Correlation between sRAGE and VCAM-1 levels in T2DM patients (*r*** **= − 0.256, *p*** **= 0.032, *n *=70).**

**Figure 4:**
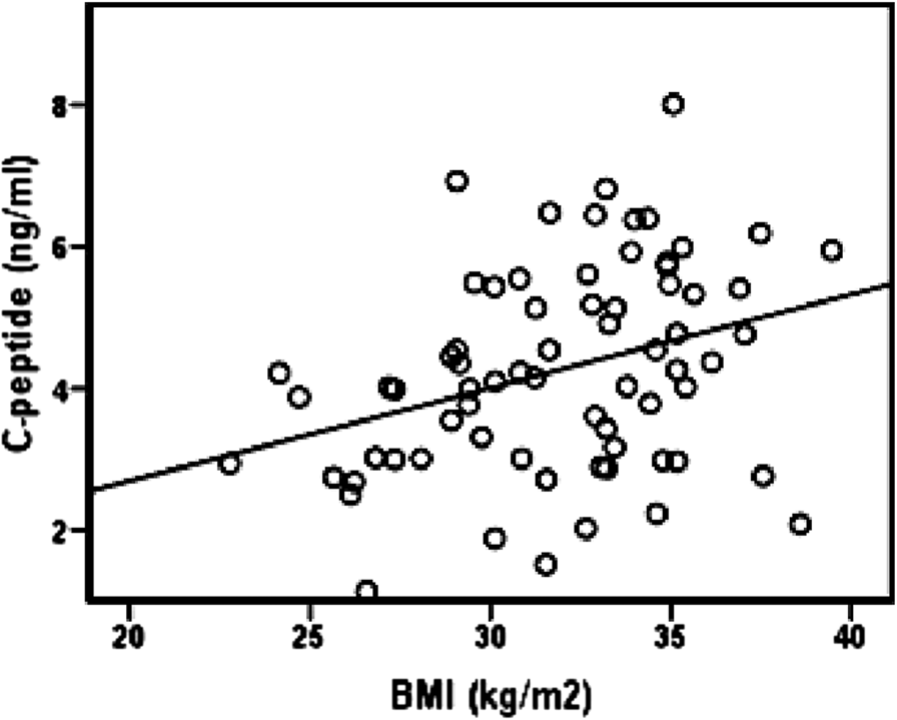
**Correlation between BMI and C-peptide levels in T2DM patients (*r*** **= 0.330, *p*** **= 0.005, *n *=70).**

Univariate analysis revealed a significant positive correlation between fasting plasma glucose and blood SOD activity in patients with T2DM (*r* = 0.377, *p* = 0.001), Figure 1. There was a significant inverse correlation between plasma ox-LDL and blood GSH in T2DM patients (*r* = − 0.412, *p* = 0.00039), Figure 2. Also, significant inverse correlation was found between sRAGE and VCAM-1 levels (*r* = − 0.256, *p* = 0.032) in T2DM patients, Figure 3. Additionally, there was a significant positive correlation between BMI and C-peptide levels in T2DM patients (*r* = 0.330, *p* = 0.005), Figure 4.

To evaluate the relationship between sRAGE and clinical and biochemical parameters, a multivariate linear regression analysis was performed in which sRAGE was included as the dependent variable. All variables listed in Table 1 were considered as independent variables. The significant predictors of sRAGE were VCAM-1 (*β* = − 0.259, *p* = 0.044) in diabetic patients (*R*^*2*^ = 0.24) and VCAM-1 (*β* = − 0.274, *p* = 0.013), and BMI (*β* = − 0.335, *p* = 0.007) in all study subjects (*R*^*2*^ = 0.257).

## Discussion

As reported by many studies [16,17], our study found decreased sRAGE level in PCD compared with healthy control. On the other hand, other studies [18,19] found higher level of sRAGE in T2DM compared with control subjects. Tan *et al.*[18] reported higher serum sRAGE levels in T2DM with proteinuria; while patients with normoalbuminuria or microalbuminuria showed no significant difference compared with normal controls. Also, Kaňková *et al.*[20] found increased sRAGE levels in patients with diabetic nephropathy (DN) than normoalbuminuric patients and reported that glomerular filtration rate was the principal determinant of sRAGE levels in diabetic subjects with DN. In the present study, our result is partially consistent with the previous studies regarding GCD group which showed no significant change compared with normal control but PCD showed significant decrease in sRAGE levels. Although, all diabetic patients in the present work had a normal kidney function, PCD had a significant decrease in sRAGE levels. Thus, poor glycemic control may decrease sRAGE level or influence its consumption. Devangelio *et al.*[16] found an increase in sRAGE levels after improvement of glycemic control and suggested that levels of sRAGE may be reduced due to excessive binding to circulating AGEs ligands. Moreover, increased ligand burden may consume all existing sRAGE and/or endogenous mechanisms that release sRAGE may be impaired [21] which may explain decreased levels of sRAGE in PCD group in the present work.

Many studies reported elevation of VCAM-1 levels in T2DM compared with control subjects [9,22,23]. In the present study, results of VCAM-1 in PCD are consistent with these previous studies. Poor glycemic control and increased glucose levels may be responsible for significant higher levels of VCAM-1 in PCD. Morigi *et al.*[24] studied the effect of hyperglycemia on VCAM-1, ICAM-1, and E-selectin in human umbilical vein endothelial cells (HUVEC) and found elevation of these parameters as well as increased leukocyte adhesion in HUVEC. Also, hyperglycemia leads to increased production of AGEs which stimulates vascular inflammation and VCAM-1 expression [25]. Thus, increase in AGEs formation in PCD due to poor glycemic control may explain significant elevation of VCAM-1 levels in this group but not in GCD in the present study. Moreover, sRAGE, which is important in capture of AGEs and prevent effect of AGEs on signaling and alteration of cellular properties [26], is significantly decreased in PCD but not in GCD which may emphasize the previous hypothesis that increased AGEs levels in PCD causes elevation of VCAM-1. It is worth to mention here that an inverse correlation between sRAGE and VCAM-1 levels was found for the first time in the present study which may suggest a protective role of sRAGE against vascular inflammation. On the other hand, Boulbou *et al.*[7] found no change in VCAM-1 levels in T2DM patients compared with healthy control. This discrepancy between studies in VCAM-1 levels may be due to differences in number of patients, glycemic control, and ethnic groups.

The present study is in agreement with previous observations [27-29] showing unchanged SOD activity in DM patients. On the other hand, some authors reported both increased [11,30] or decreased [31] SOD activity in diabetic patients compared with control subjects. Chen *et al.*[32] found a significant decrease in SOD activity in newly diagnosed T2DM patients compared with controls which increased significantly after 3-month treatment with gliclazide. It is possible that changes in SOD activity may occur in early stages of diabetes as found by Chen and coworkers. Patients in the present study had had diabetes for relatively long time and had been on long-standing hypoglycemic agents which may be a possible explanation for unchanged SOD activity in these patients. Komosińska-Vassev *et al.*[33] found significant higher SOD activities in T2DM patients and the greatest increase was found in poorly controlled diabetics with micro- and macrovascular complications. In the present study, patients were free of complications which may be another explanation for unchanged SOD activity. Treatment with different hypoglycemic agents may also influence SOD activity. Gliclazide treatment was proven to enhance SOD activity in T2DM due to its antioxidant properties, and so prevents consumption of SOD by free radicals [34]. Metformin also proved to increase erythrocyte SOD activity after 4 weeks treatment in T2DM [35].

Studies reported that NO_x_ levels decrease [36,37], increase [38,39], or unchanged [40,41] in T2DM patients compared with control subjects. Our results regarding NO_x_ levels are consistent with studies that found unchanged levels compared with healthy controls. Tatsch *et al.*[37] found decreased serum NO_x_ in T2DM and suggested that increased formation of O_2_^•–^ during oxidative stress in vascular wall may inhibit NO-mediated endothelial function by formation of peroxynitrite. In the present study, no change in SOD activities were found in the diabetic groups from controls which may protect NO^•^ from deleterious O_2_^•–^ and so, NO_x_ levels in diabetic patients did not differ from that of normal control.

Many studies reported increased LDL oxidation in T2DM [42-44]. On the other hand, Hamed *et al.*[45] found that plasma ox-LDL levels in T2DM without coronary artery disease were not significantly different when compared with healthy control. Our result is consistent with that of the previous study as our patients were free of complications. Another explanation for unchanged ox-LDL levels in diabetic patients in the present study may be due to the treatment with sulfonylureas, insulin and/or metformin which have antioxidant effects. Insulin was reported for its antioxidant effect as it suppressed LDL oxidation when given to T2DM patients [46]. Sulfonylureas have also been reported for its antioxidant effect, but this was demonstrated for gliclazide only [34,47] not all members of sulfonylurea group of compounds. Fortunately, most of patients in the present study were on gliclazide treatment which may account for unchanged ox-LDL levels in these patients. Treatment of patients with metformin may also participate in protection of LDL from oxidation. Many studies reported antioxidant effect of metformin [48,49].

Many previous studies reported decreased GSH levels in diabetic patients compared with normal control [35,50,51]. Our results are in agreement with these studies. Decreased GSH levels in DM patients may be caused by different pathways including increased activity of sorbitol pathway which depletes NADPH, so limits the reduction of GSSG to GSH; decreased activity of glucose-6-phosphate dehydrogenase in hexose monophosphate shunt in DM which generates NADPH; and passage of GSSG *via* erythrocyte membrane which inhibits its reduction to GSH [36]. Another cause for lower GSH levels in DM may be the decreased levels of amino acids necessary for GSH synthesis; L-cysteine, L-glutamate, and L-glycine [52]. Many studies have reported decreased levels of theses amino acids in DM [53,54].

Regarding C-peptide, our result is in agreement with previous study [55] reported unchanged C-peptide levels in type 2 diabetes compared with normal controls. C-peptide is secreted in equimolar amounts along with insulin and is not extracted by the liver. Therefore, C-peptide can be used as indirect measurement of β-cell function [56]. Treatment of diabetic patients with ex-ogenous insulin leads to decrease in C-peptide levels as a result of reduction in blood glucose [57]. Only 20 patients (28.6%) in the present study were treated with insulin and the rest were treated with sulfonylurea and/or metformin which may explain normal C-peptide levels in these patients. Chan *et al.*[58] found that BMI was positively correlated with C-peptide levels in type 2 diabetic patients and that obese patients had highest C-peptide levels while lean patients had the lowest level. Our results are consistent with that of Chan and coworkers as we found positive correlation between C-peptide levels and BMI. Most of patients (49/70; 70%) in the present study are obese as defined by BMI > 30 kg/m^2^[59] which may be another explanation for normal C-peptide level in these diabetic patients.

A limitation of this study is that the sample sizes were relatively small and unequal among the groups. With samples of a larger population, some of the non-significant differences may have reached statistical significance. However, the post-hoc power analysis indicated that the current number of controls and patients was sufficient to detect a significant correlation between variables. Assuming a type I error of 5% and a power of 95%, the minimum association that could be detected was r = 0.248.

Brown and Fraser [60] and Wittwer *et al.*[61] reported that sRAGE has marked individuality and variation even in healthy subjects, which may be attributed to metabolic differences that are not necessarily linked to any pathology and showed that sRAGE may be useful for intra-individual monitoring or for risk assessment for a given pathology but less efficient as a screening marker for any disease. This makes another limitation of our study especially with the present small sample sizes of the study groups. Actually, future studies with larger cohorts are needed to clarify these issues.

## Conclusion

In conclusion, in type 2 diabetic patients, free of complications, poor glycemic control decreases plasma sRAGE and increases VCAM-1 levels while good glycemic control improves these abnormalities which provides benefit to diabetic patients. The study demonstrated for first time that plasma levels of sRAGE is inversely correlated with plasma levels of VCAM-1 which may suggest a protective role of sRAGE against vascular inflammation in type 2 diabetes. Also, the use of certain hypoglycemic drugs with antioxidant properties in treatment of type 2 diabetes may provide benefit *via* reduction of oxidative stress. Further studies are needed to investigate associations between sRAGE and various relevant plasma factors as well as diabetic complications.

## Competing interest

The authors declare that they have no competing interests.

## Authors’ contributions

TMKM conceived of the study, recruited participants and reviewed the manuscript. MAA recruited patients and helped in draft the manuscript. AMAB and MOM prepared patients material for analysis, reviewed statistics and drafted the manuscript. All authors read and approved the final manuscript.

## Pre-publication history

The pre-publication history for this paper can be accessed here:

http://www.biomedcentral.com/1472-6823/13/32/prepub
